# Supplementary material for: Nanoscale redox mapping at the MoS2-liquid interface
Source: Nat Commun. 2021 Feb 26;12:1321. doi: 10.1038/s41467-021-21660-z (PMC7910562; doi:10.1038/s41467-021-21660-z)
Supplement: Supplementary file 1 — Supplementary Information [file 41467_2021_21660_MOESM1_ESM.pdf]

Supplementary Information

# Nanoscale redox mapping at the $MoS_2$-liquid interface

He-Yun Du[1,2,3], Yi-Fan Huang[4], Deniz Wong[4], Mao-Feng Tseng[4], Yi-Hsin Lee[1,5], Chen-Hao Wang[5], Cheng-Lan Lin[6], Germar Hoffmann[7,8], Kuei-Hsien Chen[1,4], Li-Chyong Chen[1,2]

[1]*Center for Condensed Matter Sciences, National Taiwan University, Taipei 10617, Taiwan*

[2]*Center of Atomic Initiative for New Materials, National Taiwan University, Taipei 10617, Taiwan*

[3]*Department of Chemical Engineering, Ming Chi University of Technology, New Taipei City 24301, Taiwan*

[4]*Institute of Atomic and Molecular Sciences, Academia Sinica, Taipei 10617, Taiwan*

[5]*Department of Materials Science and Engineering, National Taiwan University of Science and Technology, Taipei 10607, Taiwan*

[6]*Department of Chemical and Materials Engineering, Tamkang University, New Taipei City 25137, Taiwan*

[7]*Department of Physics, National Tsing Hua University, Hsinchu 300, Taiwan*

[8]*Center for Quantum Technology, National Tsing Hua University, Hsinchu 300, Taiwan*

*Correspondence and requests for materials should be addressed to L.-C.C. (chenlc@ntu.edu.tw), K.-H.C. (chenkh@pub.iams.sinica.edu.tw) or G.H. (germar.hoffmann@googlemail.com).*

## Supplementary Note 1: AFM-SECM scan mode.

A Bruker Dimension Icon for AFM with a SECM unit was used to study surfaces in a liquid cell. The SECM measurements were carried out in a PeakForce SECM module, which combines peak force tapping (PFT) imaging mode with the AFM-SECM approach. AFM measurements were performed in PeakForce controlled mode at a force of ~50 nN. PeakForce SECM imaging scans the probe under the main scan first (Supplementary Fig. 1a), then lift scan (Supplementary Fig. 1b) with 100 nm above sample surface. SECM measurements in this paper, here explicitly the recording of the feedback current were performed in the main scan mode, i.e. with the probe in close proximity of the sample[1]. The immersed sample was not externally contacted but charge neutrality was given by ion flow within the liquid cell (so called un-biased sample), which results in the measurable net charge flow occurring at the probe, which is by convention called "feedback".

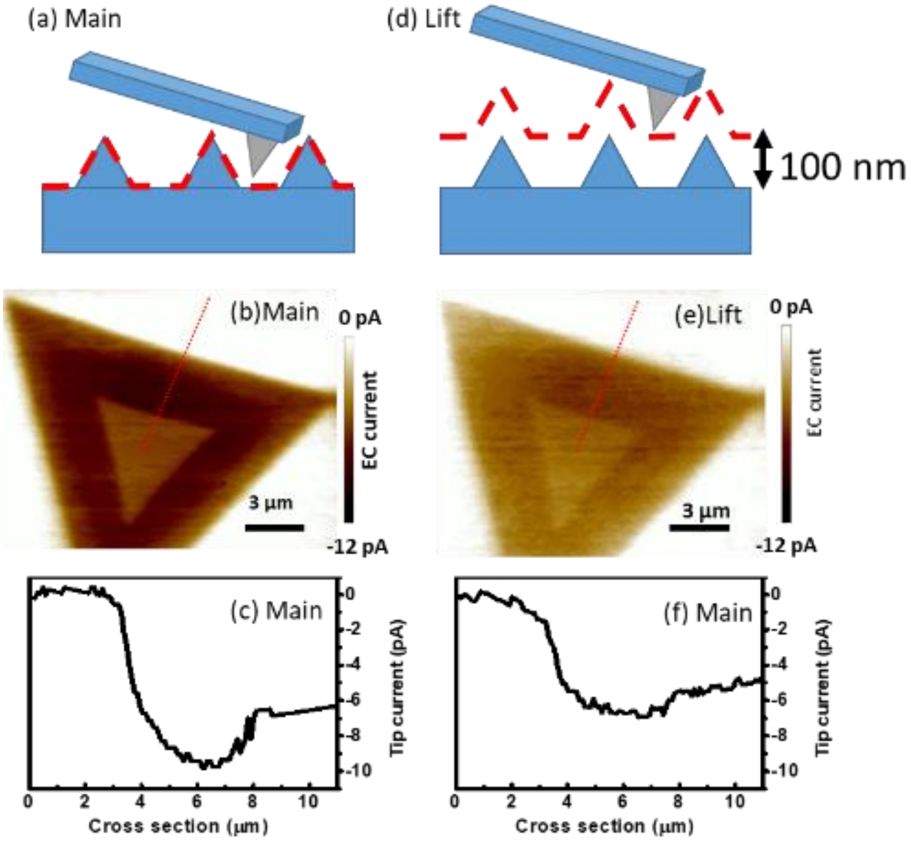


Supplementary Figure 1. Schematic diagram of AFM-SECM scanning mode.

## Supplementary Note 2

The Fc reactivity passivates the probe within hours and background currents increase up to amplifier saturation. In aqueous solution, the life time extends significantly beyond one day – but $MoS_2$ flakes detach. Therefore, we chose Fc and DmFc mediator. Whereas, the relative feedback variation, relevant for all studies here, is unaffected, a quantitative comparison of absolute feedback values within all experiments – each experiment was repeatedly performed for similar probes and samples – is prevented. Thereby, an SECM feedback signal from $MoS_2$ flakes with side lengths equal / below 3 µm was not observed (which serves as an input parameter for the relative recycling resistance calculation in Supplementary Note 3) and a quantitative feedback-size dependence can be deduced, see Supplementary Table 2, where islands of significantly different sizes were addressed within the same experiment.

Supplementary Table 2:

| Side length (μm) | 3 | 20 | 35 |
|---|---|---|---|
| Area ($\mu m^2$) | 4.5 | 200 | 612.5 |
| Tip current enhancement (pA) | Below sensitivity | 1.5 | 5 |
| Feedback normalized by flake size ($fA/\mu m^2$) | None | 7.5 | 8.2 |

**Supplementary Note 3: Relative Resistance for Recycling versus Recharging Charge Flow**

The current flow through the sample and the probe (feedback) can be interpreted within a resistance network:

$$I = \frac{const}{R}$$

and will be applied to the data presented in Fig. 3 (a, d, g). Thereby, the total resistance R is given by:

1) $R_{recharging}(area) = \frac{\tilde{R}_{recharging}}{area}$

is the total resistance experienced by the recharging process over the entire flake (area). We define $R_0 = R_{recharging}(a_r)$, with the reference area $a_r = (15\ \mu m)^2$, which approximately represents the size of the flake discussed in Fig. 3 (a, d, g) and is of similar size to Fig. 2 (b, f) of the initial size dependent study. Here, we do not distinguish between the resistance of the solution and the contact resistance (given by the Faradaic process), as both are scaling with one over area.

2) $R_{electron\ transport} \ll R_0$

We assume that the electron transport within the flake on the studied size scale is negligible; otherwise, we would expect to find a characteristic feature (depression) in SECM feedback maps of the bilayer, which we did not observe.

3) $R_{recycling} = R_r * R_0 * \mathrm{Ac}$

The relative recycling resistance, i.e. in units of $R_0$, for the recycling process. Technically, the recycling resistance also scales with one over recycling area, however, as we do the experiments at fixed probe-sample distance, the recycling area is constant and therefore, is covered within $R_r$. Ac is the activity with $Ac = Ac^{ML} = 1$ for the monolayer. It must be noted, that the assumption of a constant area is only valid when all data compared are collected with the same probe. This is the case, when we probe a single surface of different areas but limits the possibility to compare on a quantitative level data acquired with different probes. Qualitatively, it can be assumed that probes are of similar overall shape with variations covered by a very conservative approximation of values deduced from the experiments for the feedback noise and maximum size of unresolved $MoS_2$ flakes (see below).

4) $R_{recycling\ flow} \ll R_0$

The resistance for the diffusion controlled charge flow. It is justified to suspect that the charge carrier density between probe and sample is enhanced by the recycling process and confined to a small volume in comparison to the recharging process with a respectively low resistance We will in the following find, that already when we ignore the contribution of the recycling flow, the possible phase space for mathematical solutions is rather narrow and sufficient for the discussion. In so far, considering the uncertainty related to any chosen value for the recycling flow, it is not justified to assume any value beyond noting that it gives an additional contribution, which further reduces

the available phase space.

We can summarize the current dependence by a resistance network with

$$I(area) = \frac{const}{R_0 * \frac{15\ \mu m^2}{area} + R_r * R_0 * Ac}$$

From the size dependent experiments with constant $R_r$, as the recycling flux is highly localized, in comparison to the recharging flux, we can deduce the following parameters:

$$(a)\ I_{noise} < 0.1\, I(a_r)$$

The upper noise limit (see Fig. 2(h)), which determines the minimum flake size, detectable in SECM maps.

(b) $I\ ((3\mu m)^2) < I_{noise}$

The estimated current of 3 µm islands, which are the largest islands (lower limit), which could never be detected in SECM data.

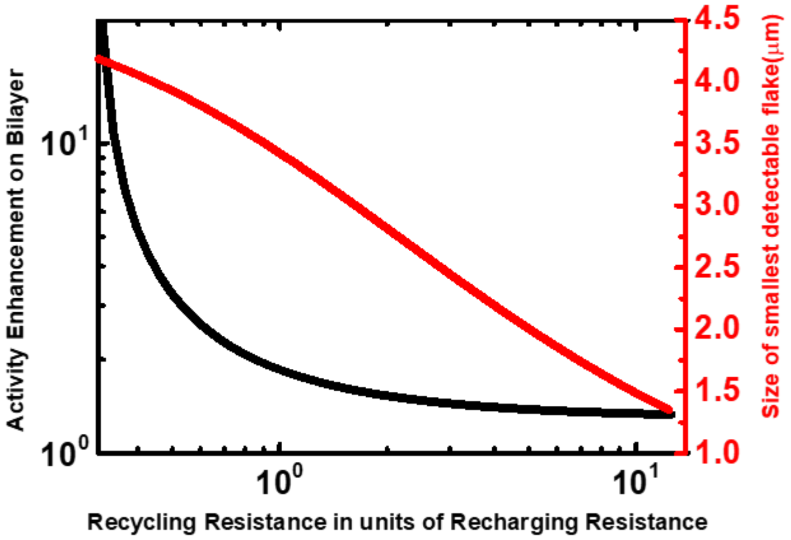


Supplementary Figure 3 (red line) shows the minimum flake size, which should be detectable within the experimental noise limit for a given ration Rr of recycling resistance versus recharge resistance. Islands with 3 µm side width are never observable at a noise level of a tenth of the feedback recorded for islands with side width of 15 µm, which is a conservative approximation to determine the upper limit of the recycling resistance. (black line) We observed a feedback enhancement of 30% on top of bilayers in comparison to the signal from the monolayer. This gives a lower limit for the relative recycling resistance $R_r$ of 0.3.

From the size dependent measurements presented in the main article, and including the full bandwidth of experimental data beyond those presented in the main article, we can estimate an upper value of the recycling resistance $R_r$. In Supplementary Fig.2 (red line), the maximum flake size (length of one of the triangular side) is plotted against $R_r$, which should within the noise limit appear unresolvable. Applied to the case studied in Fig. 3 (a, d, g), we can deduce that the recycling resistance $R_r$ is smaller than 1.6.

From the study of bilayers in comparison to monolayers, we additionally get

(c) $\frac{I_{BL}(a_r))}{I_{ML}(a_r))} \cong 1.3$

The observation of a feedback enhancement of 30% implies that the minimum relative resistance $R_r$ is 0.3. At a relative resistance of $R_r = 0.3$, the activity on the bilayer must tremendously increase of much larger than by a factor of 20 to compensate for the major impact of the dominating recharging flux resistance with no mathematical solution when the

relative recycling resistance is below 0.3.

Summary:

(a) When the current flow is entirely dominated by the recharge resistance, variations of the sample activity become unresolvable.

(b) When the current flow is entirely dominated by the recycling resistance, the relative feedback enhancement on the bilayer (or any structure) is proportional to the local activity and can be experimentally determined (see black curve) – but no flake size dependence can be expected.

(c) In the intermediate range with the recharge resistance being of the same order of the recycling resistance, a flake size dependence and activity contrast can be expected.

In the given case of Fig. 3 (a, d, g), the relative recycling resistance in units of the recharge resistance is in the range of $0.3 < R_r < 1.6$ and the activity enhancement $Ac^{BL}$ larger than 1.6 with no upper limit without additional assumptions.

## Supplementary Note 4: Optical and Raman Mapping and Raman Spectra

Raman related spectra and mapping results were acquired by a confocal Raman spectroscopic system (NTEGRA Spectra II, NT-MDT spectrum instruments) with a 473 nm excitation laser. The laser power was set at 5.0 mW to avoid possible damage by laser irradiation. The accumulation time at each spectrum spot is 1s. A 100× objective lens was used to focus the laser and collect the Raman scattered light, and an 1800 lines per mm grating was chosen for spectra acquisition.

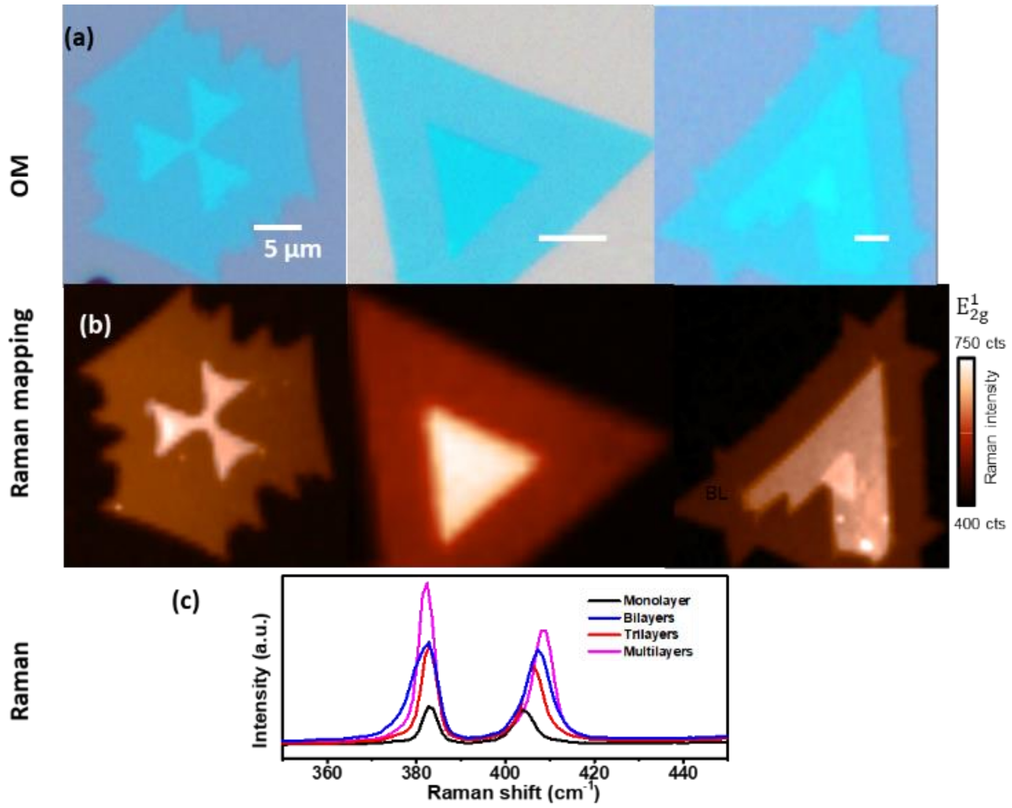


Supplementary Figure 4. (a) OM and (b) Raman mapping images of different layers $MoS_2$ flakes. (c) Raman spectra of $MoS_2$ flakes at different layer numbers with Raman peak shift between $A_{1g}$ and $E^1_{2g}$ increasing with the layer number.

**Supplementary Note 5: Relationship between mediator potentials on Ag/Ag$^+$, SHE and absolute scales.**

Supplementary Fig.5 (a) shows the reduction and oxidation peak positions of Fc and DmFc vs. Ag/Ag$^+$ reference electrode by voltammetry method. The non-aqueous Ag/Ag$^+$ reference electrode is filled with 0.01 M $AgNO_3$ and 0.1M tetrabutylammonium perchlorate (TBAP) in acetonitrile. Normalization is conducted the following way. We first measure the Fc and DmFc redox potential by the non-aqueous Ag/Ag$^+$ reference electrode. The redox potential difference between Fc/DmFc and Ag/Ag$^+$ is measured by cyclic voltammetry shown in Supplementary Fig. 5(a). Cyclic voltammetry curve is measured with the electrolyte containing 5 mM Fc (or DmFc) as redox mediator and 0.1M TBAP as supporting electrolyte in propylene carbonate. The sweep rate is 10 mV/s. The potential conversion between non-aqueous Ag/Ag$^+$ reference electrode to SHE is based on literature[2,3]. The potential difference between Ag/Ag$^+$ reference electrode in organic solvent and saturated calomel electrode (SCE) in aqueous solution is 0.337 V[2]. The conversion into potential difference between SCE and SHE, both in aqueous solution, is 0.244 V[3]. The total potential difference between non-aqueous Ag/Ag$^+$ reference electrode to SHE is 0.581 V. The conversion into the potential vs. absolute electrode potential follows[4]. The summarized comparison of the standard redox potentials for Fc and DmFc versus Ag/Ag$^+$, SHE and vacuum scale is shown in Supplementary Fig. 5 (b).

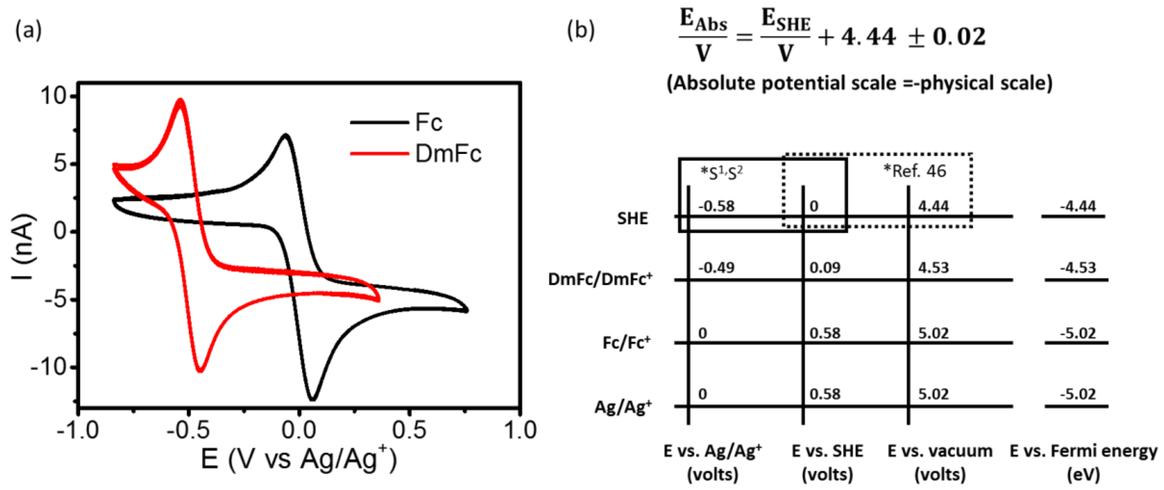


Supplementary Figure 5 (a) Cyclic voltammetry curve of 5mM Fc/DmFc and 0.1M TBAP in propylene carbonate solution (CE: Pt wire, RE: non-aqueous Ag/Ag$^+$ reference electrode). The sweep rate is 10 mV/s. (b) Relationship between standard redox potentials of Fc and DmFc in reference to Ag/Ag$^+$ electrode, SHE, and absolute scale electrode potentials.

**Supplementary Note 6: Spatial / Temporal Resolved Chronoamperometric data related to Fig. 5c of the main article and step-wise illustration of data processing in relation to current noise.**

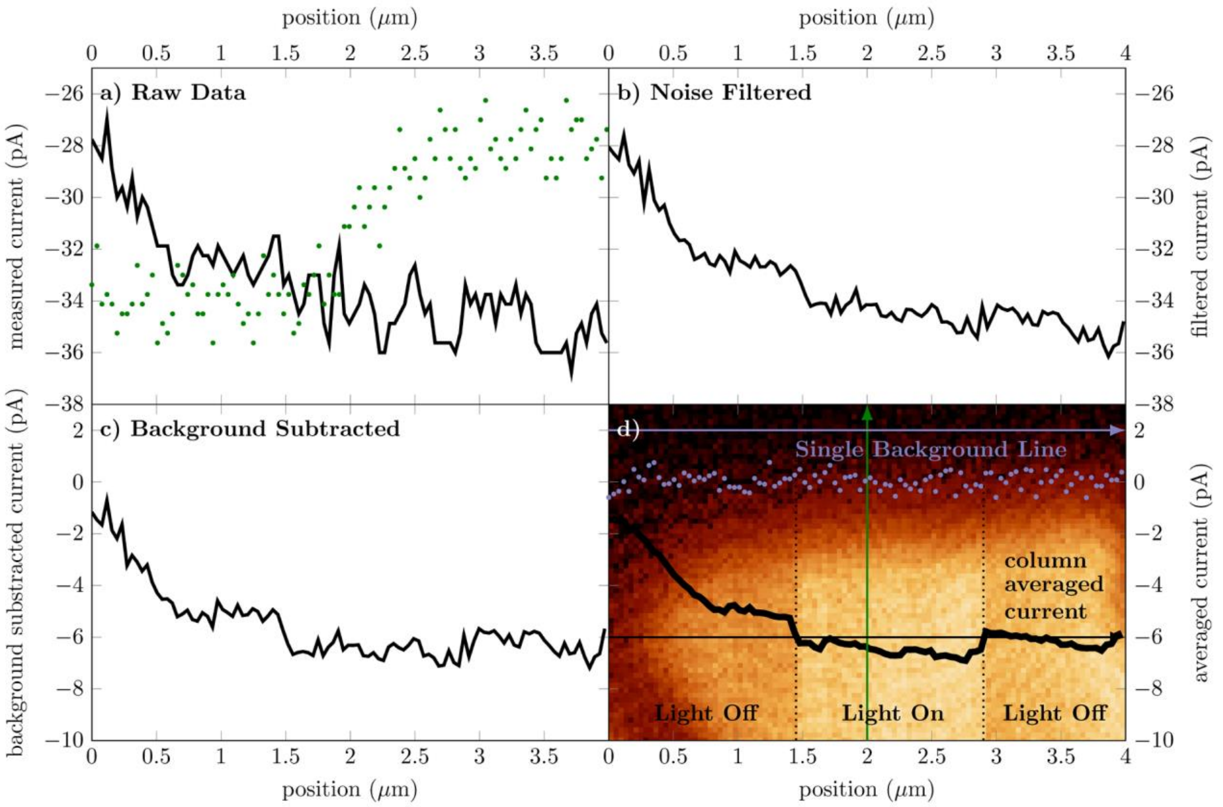


Supplementary Figure 6. a-d) show data after each processing step as obtained from line-sections at the marked lines in d) with the color encoding indicating signal from (black=) the $MoS_2$ island with time, (blue=) insulating substrate with time, and (green=) a line scan in fast scanning direction, i.e. quasi equi-temporal. The same colors are used in all subfigures. The background image in d) shows the resulting SECM map after data processing (0.6 V vs. Ag wire). Note: The single background line (blue) was acquired much above the shown image section to avoid signal interference originating from the $MoS_2$ island - but for schematic illustration parallelly projected on the depicted image area.

In Fig. 5c of the main article uncorrected Chronoamperometric I-t data, i.e. without background subtraction, are presented to qualitatively demonstrate the response of the feedback current on Light Irradiation and discussed within the framework of a respective band shift as deduced from the complete set of data gathered from different techniques. To illustrate the data processing and in the specific case of Chronoamperometric I-t data, the deduction of quantitative data and the background, we demonstrate in Supplementary Fig. 6 the entire process for the case of low-intensity signals.

Chronoamperometric I-t data are acquired at an acquisition time of 30 ms / data point and the entire data set in Fig. 5c covers 2 min 30 sec with an unambiguous modulation of the measured current with irradiation state. However, a precise quantitative background subtraction is here not feasible, as in difference to Fig. 2 / 3 the background signal originating from a temporally parallel acquisition of the current with the probe placed above the insulating substrate is not accessible. Supplementary Fig. 6 gives a demonstration from data acquired from the very same $MoS_2$ island prior to the Chronoamperometric I-t data acquisition in the scanning mode, which gives quasi equi-temporal access to island and substrate information within each line scan. The background image of Supplementary Fig. 6d) presents the SECM data after data processing, as discussed and commented on below. The entirely covered surface is larger - here, we discuss the relevant area with a $MoS_2$ island located at the bottom. Left to island in the x-position interval [0 - ~0.5 μm] the

substrate surface reappears. The next near-by $MoS_2$ island left to it starts at ~ -1μm, which is therefore here not visible. Above the $MoS_2$ island, a large substrate surface appears, which extends much beyond the chosen subimage and the signal gathered from it serves as reference. The image is scanned in fast direction in the vertical and in the slow direction in the horizontal direction at an acquisition time of 2 ms / data point, i.e. much more rapidly than for the data presented in Fig. 5c. During the acquisition time, a light irradiation was switched on / off at the indicated positions (1.45 and 2.9 μm).

Supplementary Fig. 6 a) shows cross-sections through the raw data along the lines indicated in Supplementary Fig. 6 d). Within a scanning line (green dots), the $MoS_2$ island is in comparison to the substrate clearly resolved and the respective background can be quantitatively deduced. To harness the information of the light response, the line-section over the $MoS_2$ island and respectively in the slow scan direction needs to be studied (black line). Within the precision of the raw data, which is even on this short-time scale (~1 min 45 sec) affected by the slow aging of the probe, the response of the light irradiation is not discernible as the current variation is too small.

Therefore, first electronic noise is removed from the signal by fast fourier transformation (a to b). This is a process, which needs to be very carefully applied to avoid artefacts and is ideally avoided if possible - and data covered in the main article and explicitly in Fig. 2 and 3 are unfiltered. Within the now filtered data, the weak response on the light irradiation can be traced. We still find a significant slope in the data from tip aging.
The time-resolved background signal from a terrace far away (above the chosen image range) is determined and subtracted from the filtered data (b-c). This process is equally applied to Fig. 2 and 3 of the main article. The obtained quantitative value here of ~ -6pA is the "real" feedback signal originating from the $MoS_2$ island. We note, this value can only be obtained with the acquisition of quasi equi-temporal data from the substrate. During the acquisition time of the current data set, the current drift accounts for approx. 2.4 pA (in b: ~-28.1 pA at 0 μm versus ~-35 pA at 4μm in comparison to c: ~-1.25 pA at 0 μm versus ~-5.75 pA at 4μm). At the same time, the current-changes by the light is of the order of ~1 pA and therefore a current drift needs to be fully covered for a quantitative evaluation. The resulting SECM map, i.e. after filtering and background subtraction is presented as background in d) with the light response clearly resolved at the indicated positions.

In a final step to enhance the resolution, we average vertically over a larger $MoS_2$ line scan with the result plotted in d). We find for the reference insulating substrate a nearly flat background level (blue line). However, the averaging process, which enhances the resolution on a short-time scale and gives access to a quantitative value (of a feedback current change of ~1 pA between on and off state), is strongly affected by geometrical affects (here the slope in response of the shape of the $MoS_2$ island), tip changes (jumps in the averaged lines) and the temporal evolution of the current with time after light switching. These are artefacts, which are (apart from the geometrical influence) also present in Chronoamperometric I-t curves. On the expense of a quantitative access to the background current, the temporal dynamic change of current immediately after light switching is captured with an enhanced sensitivity in contrast to the SECM mapping mode, with significant time spent on the insulating surface area and the respective tip-displacement to the far-away surface area.

## Supplementary Note 7: AFM-SECM setup for photoelectrochemical experiments

AFM-SECM setup for photoelectrochemical experiments using a white light source. ($h\nu$ = 0.3 - 1.8 eV)

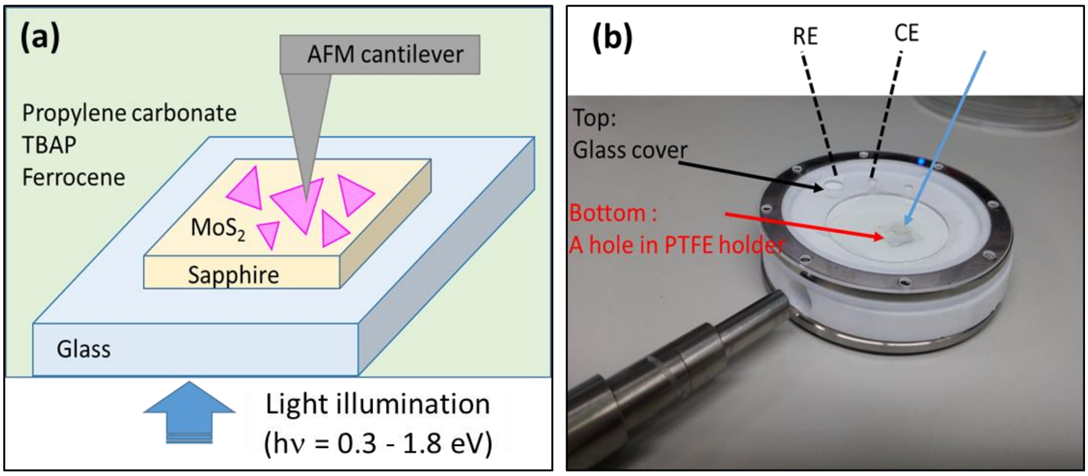


Supplementary Figure 7. (a) Schematic diagram of AFM-SECM setup during the measurement. (b)Modified photoelectrochemical cell with illumination from the bottom of the cell.

## Supplementary Note 8: The electrochemical properties of the SECM probe.

Standard AFM-SECM probes (PeakForce SECM module) from Bruker company were used in this paper. The voltammogram curves of the probe far away from the substrate (Supplementary Fig. 8a) and approach on top of $SiO_2$ substrate (Supplementary Fig. 8b) are shown.

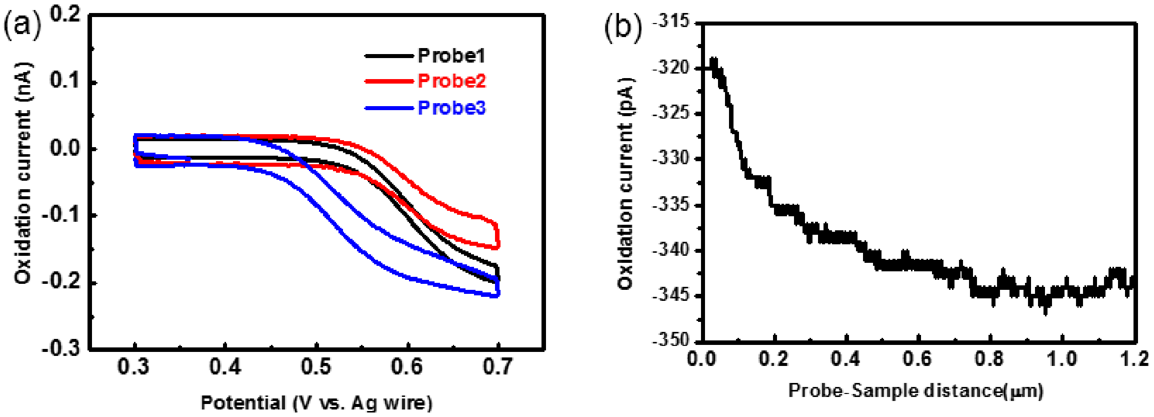


Supplementary Figure 8 (a) Cyclic voltammogram results from electrochemical test in 5 mM Fc and 0.1 M TBAP in propylene carbonate solvent. The scan rate is 50mVs$^{-1}$. (b) Approaching curve measured at $SiO_2$ surface in 5 mM Fc and 0.1 M TBAP in propylene carbonate solvent. The probe voltage is 0.6 V vs. reference Ag wire.

## Supplementary Note 9: Normalization of SECM images by cell geometry.

For normalization of the SECM feedback current, the quasi equi-temporal background current is mapped line wise on top of $SiO_2$. The probe current on $SiO_2$ (background current) in the AFM fast scan direction remains (nearly) constant but increases with time in the slow scan direction, as visible in the SECM map - see Supplementary Fig. 9(a) and

Supplementary Fig. 9(b). Supplementary Fig. 9(c) shows the SECM image after line wise normalization. We find, although the absolute current varies with time, the enhancement remains unaffected which is verified for all experiments presented.

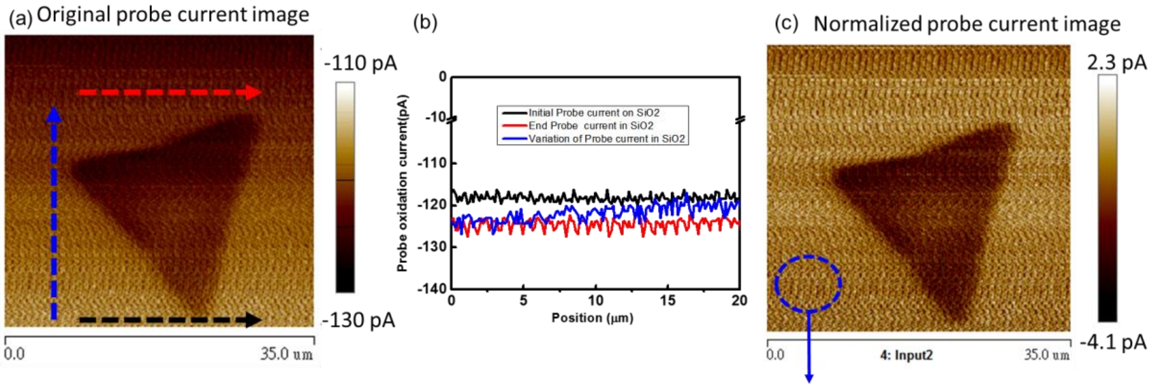


Supplementary Figure 9 (a) Raw image data. (b) Variation of the probe current from $SiO_2$ along fast (black/red) and slow (blue) scan directions. (c) SECM image data after line wise normalization by the $SiO_2$ background current.

## Supplementary Note 10: CVD growth.

$MoS_2$ was grown on a Si substrate with 300-nm thick $SiO_2$ by chemical vapor deposition (CVD) method. The schematic diagram of the CVD setup is shown in Supplementary Fig. 10. A porcelain boat with sulfur was located in the upstream region outside the furnace. For the growth, we use a sandwich structure formed of the Si substrate with the $SiO_2$ growth surface pointing towards a Mo foil on top of a quartz plate. The sandwich structure was then loaded into a 2-inch diameter quartz tube and centered in the furnace. Before $MoS_2$ growth, the system was flushed with 100 standard cubic center meter (sccm) of argon gas for 10 min. Afterwards, the sample was heated from room temperature to ~800 °C at a rate of 30 °C/min and then maintained at 800 °C for 20 min. Meanwhile, the sulfur (1g) was heated from room temperature to 160 °C at a rate of 10°C/min and carried by Ar/$H_2$ gas flow to the growth zone. The Ar and $H_2$ gas flow rates were 50 and 2 sccm, respectively. The operating pressure of CVD was 10 Torr. The growth, with $MoS_2$ flakes on top of $SiO_2$/Si wafer, ended with natural cooling of the system.

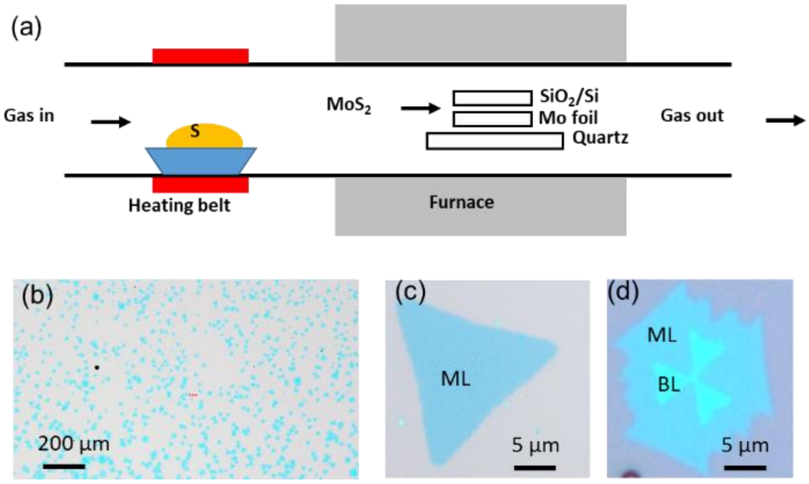


Supplementary Figure10. (a) Schematic diagram of CVD growth method of $MoS_2$ flakes. (b-d) optical microscope images at different scales of ML and BL $MoS_2$ flakes.

## Supplementary Note 11: STM and STS of $MoS_2$ at different thickness

STM and STS experiments as shown in Supplementary Fig. 11 were performed to verify the relative change of band gaps and band onsets for $MoS_2$ of different thickness. STM and STS measurements were carried out in a homebuilt UHV STM system with the microscope from RHK Technology. The base pressures of the STM chamber and preparation chamber were $6 \times 10^{-10}$ Torr and $2 \times 10^{-9}$ Torr, respectively. After the $MoS_2$/HOPG sample was loaded through the load lock chamber, it was degassed at ~250 °C for 2 h prior to scanning. The mechanically cut PtIr tip was grounded, and a bias voltage applied to the sample.

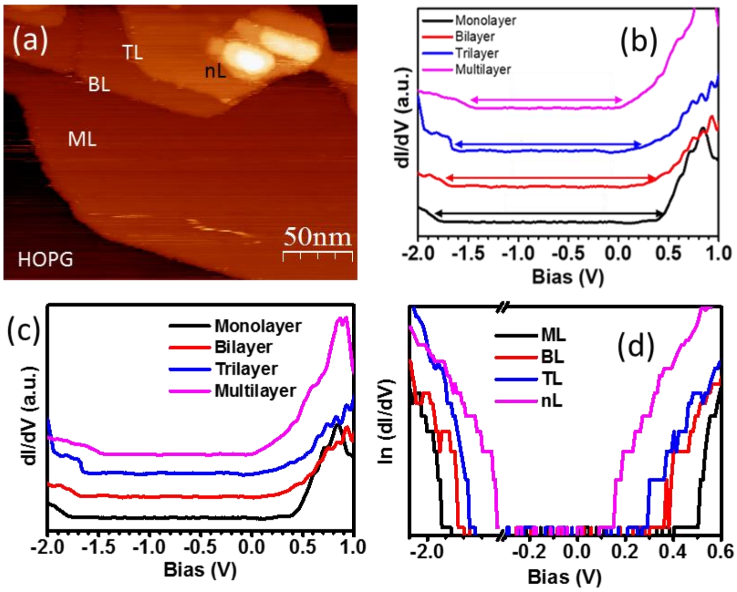


Supplementary Figure 11. STM/STS analysis of layer controlled $MoS_2$ flakes. (a) STM image (1.2 V, 49 pA) of ML, BL, TL and thicker area of $MoS_2$ sample. (b) STS data (setpoint: 1.5 V, 49 pA) of ML, BL, TL and thicker area of $MoS_2$ sample after numerical differentiation of $I/V$ curves. Valance band maximum (VBM) and conduction band minimum (CBM) are marked with arrows. (c) STM d$I$/d$V$ spectra and (d) ln (d$I$/d$V$) are showed here also.

## Supplementary Note 12: AM-KPFM

Amplitude modulated Kelvin probe force microscopy (AM-KPFM) was used to locally map the work function of $MoS_2$ after calibration of the probe work function on HOPG. Thereby, an alternating current voltage of 1.5 V at a frequency of 73 kHz was applied to a Pt/Ir coated probe. Supplementary Fig. 12 demonstrates experimental results for the monolayer and the bilayer $MoS_2$.

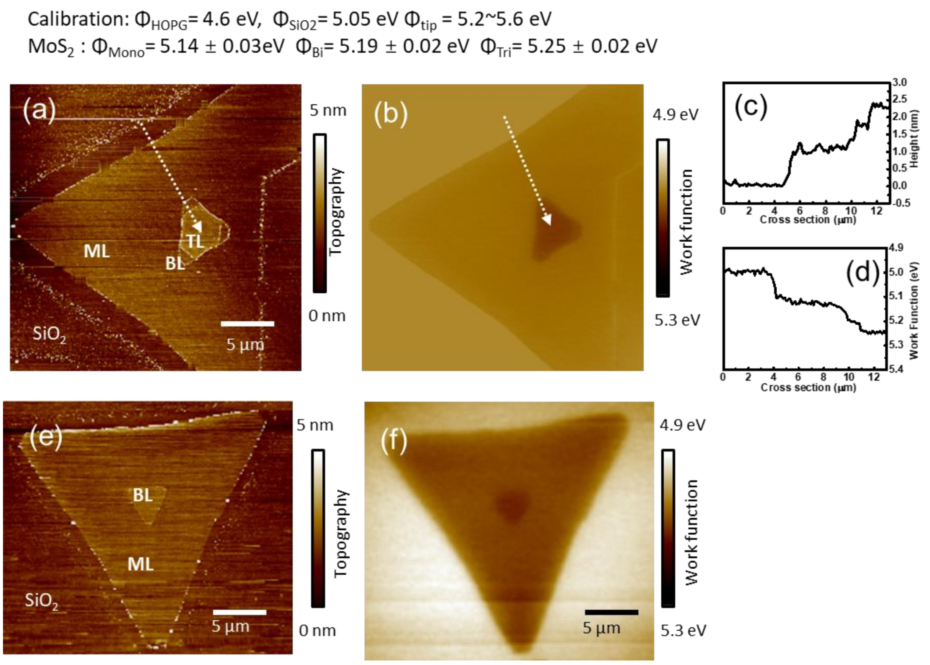


Supplementary Figure 12. KPFM analysis of layer controlled $MoS_2$ flakes. Alternating current voltage of 1.5 V at a frequency of 73 kHz was applied to a Pt/Ir coated probe. a,e) show AFM data, b,f) KPFM data for different thicknesses of $MoS_2$ (as labeled) with cross-sections given in c,d) along the indicated lines.

## Supplementary Note 13: Representative collection of work function, band gap, valence, and conduction band energies as previously reported and in comparison to values obtained from our experiments.

Supplementary Table 13

*In reference to EF / mu chemical potential

| | ML | CBM/VBM* | BL | CBM/VBM* | $\Delta E^{conduction\ band}_{BL-ML}$ | $\Delta E^{gap}_{BL-ML}$ | Reference | Technique |
|---|---|---|---|---|---|---|---|---|
| **Band gap (eV)** | 2.41 | 0.49/-1.92 | 2.21 | 0.38/-1.83 | **-0.11** | **-0.20** | This work | STS |
| | 2.40 | 0.45/-1.95 | 2.10 | 0.40/-1.70 | **-0.05** | **-0.30** | [5] | |
| | 2.15 | 0.31/-1.84 | | | | | [6] | |
| | 2.06 | 0.27/-1.79 | 1.89 | 0.27/-1.62 | **0** | **-0.17** | [7] | |
| | 2.00 | 0.75/-1.25 | 1.75 | 0.50/-1.25 | **-0.25** | **-0.25** | [8] | |
| | 1.98 | | 1.8 | | | **-0.18** | [9] | HREELS |
| | 1.88 | | 1.59 | | | **-0.29** | [10] | Photoluminescence |
| | 2.11(sapphire)<br>1.90(Au) | 0.35/-1.76<br>0.6/-1.30 | | | | | [11] | ARPES/ARIPE |
| **Work function (eV)** | **ML** | | **BL** | | $\Delta E^{work\ function}_{BL-ML}$ | | **Reference** | **Technique** |
| | 5.14 | | 5.19 | | **0.05** | | CVD (this work) | KPFM |
| | 4.93 | | 4.88 | | **-0.05** | | CVD[12] | |
| | 4.49 | | 4.54 | | **0.05** | | Exfoliate[13] | |
| | 5.15 | | 5.25 | | **0.1** | | Exfoliate[14] | |
| | 4.64 | | 4.69 | | **0.05** | | Exfoliate[15] | |
| | 4.36(ambient)<br>4.04(UHV) | | | | | | [16]<br>[16] | |
| | 4.93($SiO_2$/Si)<br>5.10(Au) | | | | | | [12]<br>[12] | |

The electronic bandgaps (reported and our data) of $MoS_2$ mono- and bilayer determined vary by ±0.2 eV in STS. Thereby, the absolute values depend systematically on the used numerical processes, whereas relative changes with layer when compared based on the same numerical process, are unaffected by the systematic variation. The same holds for the comparison of absolute values and relative changes of the valence band maximum (VBM) and the conduction band minimum (CBM) from different labs. Quantitatively and relevant for our analysis, we find similar values in the obtained changes (ΔE) of Band Gap Energies and Conduction Band Shifts.

The optical bandgap of ML $MoS_2$ observed by PL (~1.9 eV) is usually lower than the electronic bandgap observed by STS (above 2 eV) which is due to the formation of an exciton[5].

**STS**

1. Most of the STS results (and all listed here) of $MoS_2$ flakes show n-type semiconductor behavior both in monolayer and bilayer. This is related to the intrinsic S vacancy on the $MoS_2$ surface. We also confirmed this by STS results both in monolayer and bilayer area.
2. We observed a band gap decrease of 0.2 eV from monolayer to bilayer $MoS_2$. Other labs also observed the variation ranging from 0.17 to 0.30 eV. The variation among different labs might be related to different surface properties of $MoS_2$ due to different CVD growth conditions. This might also lead to the different variations of CBM between ML and BL. The decrease tendency of CBM position is qualitatively the same in those references in comparison to our results.

**Work function**

1. KPFM is the most popular and standard technique to measure the $MoS_2$ surface potential and, by conversion, the work function. However, this measurement is highly dependent on environmental conditions and substrates. Respectively, Supplementary Table 13 reveals a large variation of the $MoS_2$ work function between different labs, growth methods, and humidity.
2. Whereas the reliability of absolute values suffer under the systematic experimental conditions, relative work function differences between ML and BL can be measured during the same sample scan (at the same time) and are rather unaffected by the change of conditions.

**Supplementary References:**


1 Nellist, M. R. et al. Atomic force microscopy with nanoelectrode tips for high resolution electrochemical, nanoadhesion and nanoelectrical imaging. *Nanotechnology* **28**, 095711 (2017).

2 Larson, R. C., Iwamoto, R. T. & Adams, R. N. Reference electrodes for voltammetry in acetonitrile. *Anal. Chim. Acta.* **25**, 371-374 (1961).

3 Donald T. Sawyer, A. S., Julian L. Roberts. Electrochemistry for Chemists,( Wiley-VCH Verlag GmbH & Co. KGaA, 1974).

4 Trasatti, S. The absolute electrode potential: an explanatory note. *J. Electroanal. Chem.* **209**, 417-428 (1986).

5 Huang, Y. L. et al. Bandgap tunability at single-layer molybdenum disulphide grain boundaries. *Nat. Commun.* **6**, 6298 (2015).

6 Chiu, M.-H. et al. Determination of band alignment in the single-layer $MoS_2$/$WSe_2$ heterojunction. *Nat. Commun.* **6**, 7666 (2015).

7 Trainer, D. J. et al. Inter-Layer Coupling Induced Valence Band Edge Shift in Mono- to Few-Layer $MoS_2$. *Sci. Rep.* **7**, 40559 (2017).

8 Koós, A. A. et al. STM study of the $MoS_2$ flakes grown on graphite: A model system for atomically clean 2D heterostructure interfaces. *Carbon* **105**, 408-415 (2016).

9 Dileep, K., Sahu, R., Sarkar, S., Peter, S. C. & Datta, R. Layer specific optical band gap measurement at nanoscale in $MoS_2$ and $ReS_2$ van der Waals compounds by high resolution electron energy loss spectroscopy. *Int. J. Appl. Phys.* **119**, 114309 (2016).

10 Li, X. & Zhu, H. Two-dimensional $MoS_2$: Properties, preparation, and applications. *J. Materiomics* **1**, 33-44 (2015).

11 Park, S. et al. Direct determination of monolayer $MoS_2$ and $WSe_2$ exciton binding energies on insulating and metallic substrates. *2D Mater.* **5**, 025003 (2018).

12 Li, F. et al. Layer Dependence and Light Tuning Surface Potential of 2D $MoS_2$ on Various Substrates. *Small* **13**, 1603103 (2017).

13 Ochedowski, O. et al. Effect of contaminations and surface preparation on the work function of single layer $MoS_2$. *Beilstein J. Nanotechnol.* **5**, 291-297 (2014).

14 Choi, S., Shaolin, Z. & Yang, W. Layer-number-dependent work function of $MoS_2$ nanoflakes. *J. Kor. Phys. Soc.* **64**, 1550-1555 (2014).

15 Feng, Y. et al. In situ visualization and detection of surface potential variation of mono and multilayer $MoS_2$ under different humidities using Kelvin probe force microscopy. *Nanotechnology* **28**, 295705 (2017).

16 Lee, S. Y. et al. Large Work Function Modulation of Monolayer $MoS_2$ by Ambient Gases. *ACS Nano* **10**, 6100-6107 (2016).
